# The NBS Vision System in the AMRF

**DOI:** 10.6028/jres.093.145

**Published:** 1988-08-01

**Authors:** Marilyn Nashman, Karen J. Chaconas

**Affiliations:** National Bureau of Standards Gaithersburg, MD 20899

**Keywords:** Automated Manufacturing Research Facility, computer vision, hierarchical design, image processing, Material Handling Workstation, multiprocessor, real-time, Real-Time Control System

## Abstract

This article describes the NBS Vision System developed by the Sensory Intelligence Group of the Robotics System Division which is used in the Automated Manufacturing Research Facility (AMRF). It discusses the objectives of the Vision System and its applications in the factory environment. Since the Vision System is a multi-processor system, each process is described according to its position in the vision hierarchy as well as to its particular logical and computational functions. The interfaces between the individual processes of the Vision System and the interfaces between the Vision System and other AMRF systems are described. AMRF documentation packages describing the Horizontal Workstation, the Real-Time Control System and the Material Handling Workstation are available from the Center for Manufacturing Engineering.

## Introduction

The use of real-time sensory feedback in the manufacturing work place adds the dimension of intelligence to automated tasks. With the use of specially mounted cameras, visual information extracted from a scene is made available to the appropriate Automated Manufacturing Research Facility systems. This information enables robot control systems and planners to react appropriately to the tasks and objects in their environment.

The NBS Vision System is an industrial parts recognition system which operates in real-time. It is a model based system which recognizes parts by matching an input image with a set of predefined models of parts. Models of a known set of industrial parts, computed in advance, reside in a database and are used to identify each instance of an object and to specify its position and orientation relative to a fixed camera configuration. This system is particularly suited for a factory application because the number of parts to be identified is small, the parts can be exactly specified with known tolerances on the dimensions, and the features of the parts, e.g., corners, holes, etc., are distinctive.

The NBS Vision System serves two sub-systems of the AMRF: it is used both in the Horizontal Workstation (HWS) and the Material Handling Workstation (MHWS). In the HWS, vision is used to confirm or reject the identity of a machine part and to compute the position and orientation of confirmed parts. In the MHWS, vision is used to verify the contents of trays being delivered between workstations.

## Physical Description of the NBS Vision System

**Figure 1:**
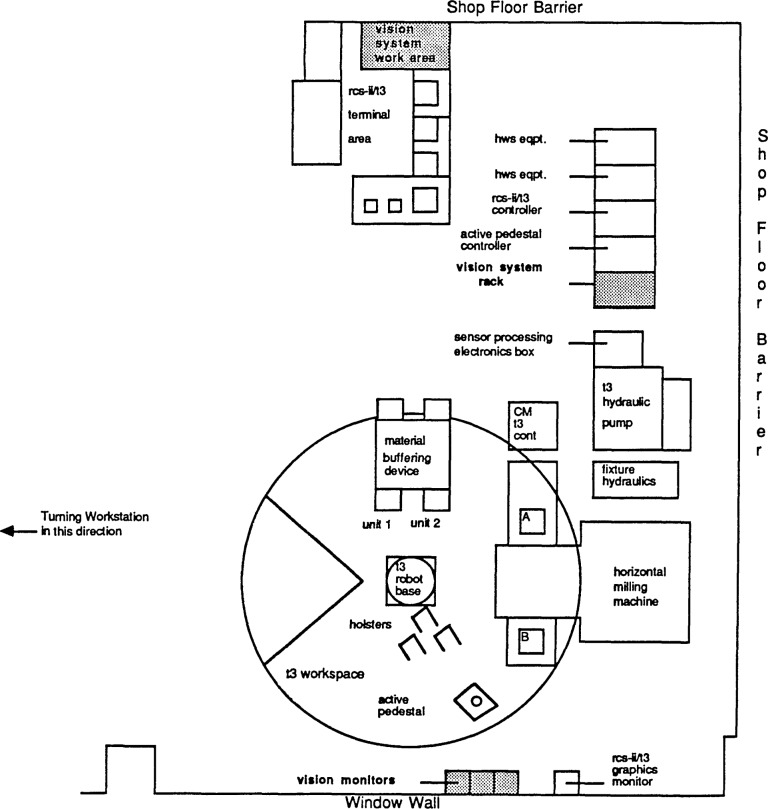
AMRF shop floor.

The NBS Vision System accepts images from a camera mounted on the wrist of a manipulator at the HWS as well as a stationary camera mounted on an inspection gantry in conjunction with the MHWS. Figure 1 describes the AMRF floor plan and the physical location of the NBS Vision System in the factory. Both cameras are electronically attached to a hardware circuit that converts the incoming signals into black and white images and compresses that information as it is read. All visual information is extracted by analyzing this resultant binary image.

**Figure 2:**
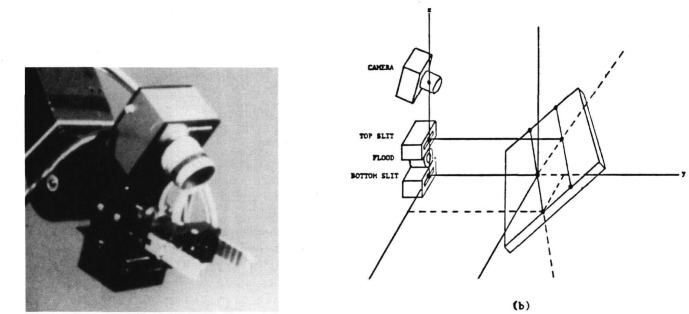
(a) Camera and light source mounted on the manipulator. (b) Relationship between the camera and the light sources.

The manipulator camera used at the HWS is fixed on the robot wrist. Below the camera, at a known distance from it, a light source is also attached to the wrist. The light source is able to generate two kinds of light flashes. The first is a flood flash that illuminates the whole field of view of the camera while the second light source generates two parallel planes of light which illuminate those points in the field of view that intersect the planes of light (fig. 2) [1].

**Figure 3:**
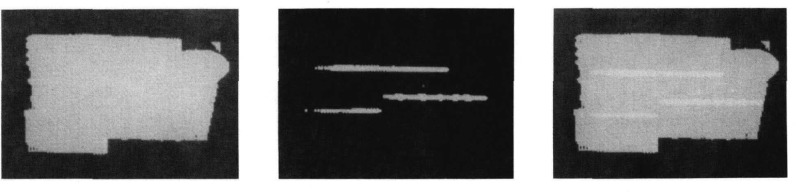
Left: Binary image of an object as seen under flood lighting. Center: Object as seen using the plane of light. Right: The two images superimposed.

The flood flash is used to find shape features such as the outlines of objects in two dimensions. The structured light is used to compute the pitch and yaw orientations of simple geometric surfaces and the range to objects. A combination of structured light and flood flash enables the system to extract elevation, azimuth, and roll orientation of the illuminated surfaces (fig. 3) [2].

The camera mounted on the inspection gantry is provided with flood lights rather than a flash. Since this is a fixed configuration, the range to the objects in the scene is always known and thus only two dimensional shape features are required for analysis.

## Overview of the NBS Vision System

**Figure 4:**
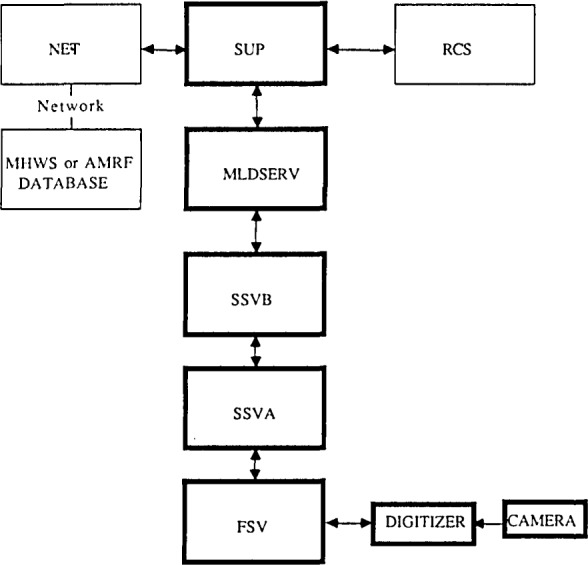
NBS vision system.

The NBS Vision System is designed in a hierarchical manner: commands from both the MHWS and the Realtime Control System (RCS) operating in the HWS are decomposed into lower-level tasks and executed by appropriate levels of the system. There are currently six independent processes operating asynchronously which analyze and extract information from an image scene. Each process resides on its own microprocessor board and communicates with other processes via a pre-defined common memory block. System routines have been developed to insure the integrity of this data transfer (fig. 4).

Three of the processes operate in a bottom-up mode to read an image and globally extract information from it. These processes are First Stage Vision (FSV), Second Stage Vision A (SSV A), and Second Stage Vision B (SSVB). A fourth process, the Multi-Level Database and Server (MLDSERV), acts in a top-down mode using its database of models to identify specific objects in a scene. The Supervisor Process (SUP) monitors Vision System activity and receives commands from other AMRF systems via the Netboard (NET) processor. Commands for vision processing are passed from SUP to the Server which determines what a command is requesting and how to answer it.

There are currently four types of requests to which the Vision System responds. In all cases, information is supplied only when commands from either the RCS or the MHWS are received. The three questions posed by the RCS include a request for the position of the centroid of the object in view; a verification of a specific factory part in a scene, and if verified, the position and orientation of that part; and lastly, the computed range to the surface of the object in view. The answers supplied by the Vision System enable the RCS to compute the required robot motion to position the manipulator and plan a trajectory for picking up the verified object at the optimum grasp point.

The fourth type of question to which the Vision System responds is posed by the MHWS and is a request for verification of the contents of a tray of parts being delivered between AMRF workstations. Through the AMRF database, the MHWS supplies information regarding the intended contents of a particular tray, e.g., the number of parts contained in the tray and the sectors of the tray in which particular parts are expected to be located. By analyzing the image of the tray and comparing the actual tray's contents with the internal model database, this information is either confirmed or rejected. A tray which fails verification is not processed further.

## Hierarchy of the Vision System

First State Vision is the lowest level in the vision processing hierarchy and acts as the interface between the camera hardware and the Vision System. It receives requests for raw image information from Second Stage Vision A and translates those requests into camera hardware commands. It then reads a compressed form of the camera image and transfers it to the appropriate common memory locations.

**Figure 5:**
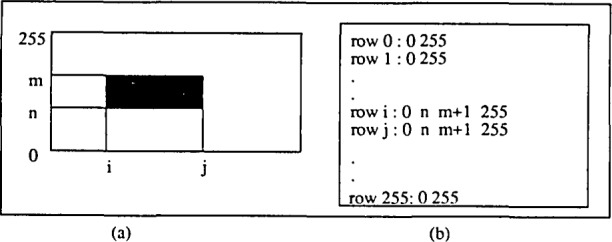
Data representation. (a) Binary image. (b) Run-length representation.

FSV controls the commands sent to the frame-buffer, which converts analog signals from the camera into digital intensity information. The resultant image is a rectangular array of image data or pixels which are assigned integer grey levels depending on their measured brightness. Thresholding converts these grey levels to two colors, black or white, depending on whether an individual pixel value is less than or greater than the given threshold value. The binary image is converted to run-length encoded information, a compressed data representation which records only transitions from black to white or white to black (fig. 5), and is read and stored by FSV.

SSVA receives its commands from the next higher level of the hierarchy, SSVB. It waits for FSV to read in an image and then reads the run-length encoded data from a predefined common memory area. SSV A performs different operations on the incoming data depending on the kind of picture (flood or structured light) that has been requested.

On receiving picture data from a floodlit image, SSV A performs a connected components analysis [7] and constructs a list of the objects (blobs) in the image. It also computes various properties of each blob such as the area, centroid, moments, etc. and encodes all points on the boundary. When the image results from using parallel planes of light, SSV A constructs groups of connected curve segments [7]. The segments correspond to single curves or to pieces of curves starting where a curve splits and ending when it ends or merges with another curve. The results of these computations are passed to SSVB by storing the structures into another common memory block.

SSVB receives commands from the first level of the Multilevel Database Processor (MLDSERV) and passes them down to SSVA. Upon receiving the data structure of blobs or curves in the image, it performs feature analysis on each object in the image. For floodlit images, this involves finding the corners, principal axis, number of holes, and perimeter of each object. For structured light images, each segment is described in terms of a polynomial and the endpoints. SSVB sends the list of objects and a set of structures describing the features to MLDSERV and then waits until another command is received.

In addition to acting as the Vision System database, MLDSERV is also the module which performs recognition and matching tasks. As a database, it stores the results of the lower-level processing in structures appropriate for the upper-level recognition algorithms. It also contains a database of statistical information which has been computed a priori describing attributes of all parts to be accessed by the RCS and the MHWS. The Server is the portion of MLDSERV which interprets commands received by SUP and formulates an answer to the questions posed. Its main activities are concerned with recognizing objects and extracting relevant information from the database. In addition, this module performs some of the range and position computations and is responsible for driving the graphic output displays.

SUP is considered to be the “brains” of the Vision System. It accepts commands from either the RCS or the MHWS, interprets them, translates them into a form suitable for the Vision System, and initiates the command sequence in the Vision System. If a poor quality image is detected, SUP tries to adjust the camera parameters to improve the picture and then repeats the process. If there are no processing errors, it sends the appropriate status and answers to either the RCS or the MHWS. In addition, SUP checks that each of the vision levels is working and monitors the terminal keyboard to service interactive features useful for providing information to the human operator.

## System Interfaces

The Vision System processes interface with each other using a common memory area governed by a file system. The interprocess communications are handled using a library of system routines developed at NBS [6]. The purpose of the library is to ensure data integrity of communications and to allow for asynchronous communications between processes. Blocks of common memory, called “files”, which are accessible to each vision process are defined. Flags associated with each file record the current state of each file. The state of a file includes information about the process which owns the file, the status of the file (open or closed), the last process which accessed the file, and the system time of last access. Only those processes granted read and/or write permission can access a file, and a process can only access a file if it is closed. The concept of opening and closing files is analogous to UNIX file system calls [4].

Communications between the RCS and the Vision System are handled using a microprocessor communications board. A master-slave relationship is represented on the board to describe the relationship between the two systems and to indicate the direction of communication. Communication is initialized by the RCS and protocols and command structures are used to allow the RCS to request service and to receive the necessary information from the Vision System.

The MHWS and the Vision System communicate using the VAX common memory mailbox system [5]. Mailbox areas are established when network communications are made to provide for message passing between systems. The Vision System queries the mailbox command area for a change in sequence number to detect when a tray verification request has been sent. Upon completion of the recognition task, a status report is sent to the MHWS mailbox to close the transaction.

## Conclusion

The NBS Vision System is currently being used in the AMRF to provide sensory information to the Realtime Control System and the Material Handling Workstation. Inputs are interpreted by a hierarchically organized group of microprocessors. The system uses knowledge of object prototypes to provide real-time feedback to the robot control system and to verify the contents of trays of machine parts. Planes of light and flood light sources are used to quickly obtain the six degrees of freedom of an object relative to the robot. The Vision System is then able to interpret an object’s 2-D outline in 3-space and to locate its centroid and principal axis. This information is used to guide the robot’s actions and trajectory as well as to identify specific parts.
